# Synthetic gel structures in soils for sustainable potato farming

**DOI:** 10.1038/s41598-019-55205-8

**Published:** 2019-12-09

**Authors:** Andrey Smagin, Nadezhda Sadovnikova, Marina Smagina

**Affiliations:** 1 Lomonosov Moscow State University, Leninsrye gory 1-12, 119991 Moscow, Russia; 2 Institute of Forest Science of RAS, Sovetskaya 21, 143030 Moscow region, Uspenskoe Russia

**Keywords:** Pathogens, Plant biotechnology, Environmental biotechnology, Agroecology, Hydrology

## Abstract

Anti-pathogenic protection of potatoes remains one of the most pressing problems of sustainable agronomy and plant protection. For this purpose, we propose to use a new type of synthetic hydrogels filled with amphiphilic recipients (dispersed peat, humates) and modern plant protection products. We assumed that the introduction of swollen gel structures into the rhizosphere of potatoes will allow us: to optimize the water supply and productivity of potatoes; to protect the fertile layer and potato tubers from the main pathogens; to fix modern plant protection products in the rhizosphere, keeping them from leaching and entering the environment. Preliminary laboratory experiments tested the anti-microbial activity of gel structures, as well as their water retention, dispersity and hydraulic conductivity with subsequent computer modeling of the water exchange and root uptake in the system of “soil-gel-potato”. Field trials were carried out in humid (European Russia) and arid (Uzbekistan) conditions under the atmospheric precipitation and irrigation on different soils and potato varieties with instrumental monitoring of environment, potato growth and quality. All experimental results confirmed the high efficiency of water-accumulative and plant protective synthetic gel structures. Their usage sufficiently (up to 6–15 t/hct) increases the potato yield with 1.3–2 times water saving, complete retention of agrochemicals in the rizosphere, and its actually total protection against major potato pathogens, including late blight (Phytophthora infestans).

## Introduction

Modern sustainable and environmentally friendly agriculture is looking for new technologies that allow us to obtain high yields of quality food products with minimal risk of environmental pollution, along with effective use and preservation of natural resources. One of the promising trends here is the development of agrochemical delivery systems to reduce pollution and health hazards^1–3^. In such a system, a pesticide, fertilizer or other bioactive agent is incorporated into a carrier, generally a polymeric material or a solid-phase adsorbent. It minimizes the impact of these harmful chemicals to the environment by reducing losses due to leaching, volatilization and biodegradation, and thereby, can potentially maintain biological efficacy of an active ingredient for a given time at a fixed point in space^2,3^.

In the framework of this direction, our research presents the results of an innovative development using the synthetic gel structures (SGS) to aid soil in supplying plants with water, dissolved substances and simultaneously protecting the rhizosphere from pathogens. For the first time, as the controlled-release formulations, we offer combined polymer-adsorbent SGS based on acrylic hydrogels filled with amphiphilic solid-phase components, trace elements and modern plant protection products (PPP) in the form of silver ions, nanoparticles, and synthetic azoxystrobin fungicide. Unlike the widely used natural hydrophilic polysaccharides (cellulose, starch, alginate, dextran, pectin, chitosan) as controlled-release formulations of agrochemicals^2,3^, the SGS based on acrylic polymers filled with the amphiphilic nature components are more resistant to soil biodegradation (a half-life (*T*_0.5_) by 3–5 years or more^4^, potentially minimizing the risk of leaching mineral fertilizers and pesticides from SGS. Most systemic pesticides have a half-life of less than 1 year; usually 30 < *T*_0.5_ < 90 days for pesticides used to protect plants within a single cropping season^5^. Therefore, they must be guaranteed to disappear before the gel carriers are decomposed by soil microorganisms. Similarly, water-soluble fertilizers and trace elements will be absorbed by the roots of plants before the biodegradation of gel structures allows them to leach from the rhizosphere.

Our study, preceded by the laboratory experiments and technological simulations in HYDRUS-1D software^6^, for the first time tests the effectiveness of new SGS in practice, aiding in the development of more sustainable cultivation and protection of potatoes directly in the soil. Most known PPP are targeted at predominantly surface treatment (spraying) and pest control of vegetative and generative plant’s organs located above the soil. In this case, the underground phytomass remains practically unprotected. Therefore, it is often affected by pathogens that inhabit the rhizosphere, resulting in great yield damage, especially on farms and seed nurseries where row crops in the form of tubers and root vegetables are cultivated year after year without rotation^7^. Under such conditions, the soil may be subject to constant infection by pathogenic microflora (fungal and bacterial rot, oomycetes) that negatively affects seed material, and later the crop itself. In the Russian Federation, annual potato crop losses are about 20–30% depending on the cultivar, and in some years exceed 50% caused by similar pathogenic agents^8^. Worldwide average losses are estimated to be 8–10% - a several billion dollar loss^9^. Taking into account the great economic significance of the problem of anti-pathogenic soil protection for potato farming and the possibility of solving it on the basis of agrochemical delivery systems, in 2016 we started a project “Protective gel compositions for combating negative biological and edaphic factors of potato growing”, supported by the Russian Scientific Foundation. This publication summarizes the main results of a 3-year long project developing and experimentally testing a new controlled-release system with synthetic gel carriers to protect and improve potato rhizospheres in humid (European territory of Russia) and arid (Uzbekistan) conditions under atmospheric precipitation, drip and furrow irrigation.

## Materials and Methods

### Synthetic polymer hydrogels

The new materials were produced at the Ural Chemical Factory (Russian Federation, Perm) under the trademark “Aquapastus” according to our patented technology of synthesis of filled acrylic hydrogels (Patent RU № 2639789 https://www1.fips.ru/publication-web). These products included various compositions of acrylic copolymers based on acrylamide and acrylic acid salts, filled by wastes of biocatalytic production of acrylamide, salts of humic acids in an aqueous paste as well as dispersed peat with additives of ionic silver. Methylene-bis-acrylamide was used as a crosslinking agent. The water absorption of new products by swelling in distilled water varies from 340 to 500 kg/kg for granules with in sizes near 1 mm sizes. The “Aquapastus”-11 (A11) hydrogel is the base co-polymer of acrylamide and ammonium acrylate filled (28%) by solid wastes of a biocatalytic production of acrylamide as a mixture of microbial cells, cell agglomerates and filtroperlit. The formulation “Aquapastus”-11H (A11H) includes, in addition to biocatalytic wastes (12%), humates of potassium and sodium amounting to 8% of dry matter. Its modification A11HMZ hydrogel is similar to the previous one, but with the addition of magnesium and zinc (the trace elements of mineral nutrition), 0.4% in terms of metals. The last two compositions A22 and A22Ag, along with the co-polymer of acrylamide and sodium acrylate, contain a finely dispersed peat as a filler (23.5%) and 0.1–1% additives of ionic silver. In laboratory experiments, the new products, described above, were compared with the well-known “Aquasorb” brend, manufactured by SNF-group (https://www.snf-group.com). It is a superabsorbing anionic polymer in the form of crosslinked copolymers of acrylamide and potassium acrylate, characterized by a maximum degree of swelling of at least 500 kg Н_2_O/kg for a granule size of 0.2–0.8 mm (Aquasorb 3005KM).

### Water-accumulative and controlled-release protective gel’s compositions

The gel structures were obtained by free 24-hour swelling of the hydrogels in pure water or in the solutions containing water soluble or suspended PPP, with a “dry gel: liquid phase” ratio equal to 1:100. As the PPP, we used a chemically pure AgNO_3_, colloidal silver, and “Quadris” sythetic fungicide with azoxystrobin produced by Syngenta (Switzerland, Basel; https://www.syngenta.com). This fungicide is one of the few pesticides for intra-soil application that are allowed in the Russian Federation. The colloidal silver is an experimental product “Zeroxxe “ of AgroChimProm GC (http://tdahp.ru/en/) that contains silver nanoparticles with a size of 10–70 nm superficially modified with environmentally safe biodegradable amphiphilic surfactant (tallow amphopolycarboxyglycinate-stabilizer)^10^.

A laboratory assessment of the protective properties of the gel compositions was performed on a potato-dextrose agar medium with addition of the PPP in a wide range of concentrations from 10 to 1000 ppm (per water mass). For the field experiments, the controlled-release SGS were prepared in 50-liter plastic containers by a similar way with free swelling in aqueous solutions containing various PPP in a range of the concentrations from 20 to 500 ppm. A laboratory study of water-retention capacity, dispersity and hydraulic conductivity was carried out on pure gels and mineral-gel compositions of 0.05 to 0.3% dry hydrogels by weight of the mineral substrates, represented by a medium-grained and fine-grained quartz sands.

### Laboratory experiments

#### Laboratory analysis of SGS protective antimicrobial properties

Protective fungicidal and antibacterial properties of the hydrogel compositions were tested using isolates of late blight (Oomycete *Phytophthora infestans* (Mont.) de Bary) and black leg of potatoes (*Pectobacterium atrosepticum* (van Hall 1902)) obtained from the State collection of phytopathogenic microorganisms in All-Russian Research Institute of Phytopathology (http://vniif.ru/vniif/structure/collection). We used collection strains isolated in 2003–2007 from the Red Scarlett and the Sante potato varieties in the Moscow and Krasnodar regions of the Russian Federation. To identify the strains, a set of differentiator varieties including 22 genotypes from R1 (CIP N800986) to R11 (CIP N800996) obtained from the International Potato Center (CIP, Peru, https://cipotato.org) was used before the isolate was placed in the collection. A preliminary test of the pathogens at the Institute of Phytopathology confirmed their virulence in relation to susceptible potato varieties. The main experiment was carried out at the optimum temperature (25 °C) using potato-dextrose agar medium (PDA) for the growth of pathogenic microorganisms (grated potato 200 g; dextrose 20 g; agar 20 g; tap water 1000 ml, pH 6.5–7.0). In the SGS trials, the hydrogels and agar were mixed in a 1:1 ratio with a dose of 10 g of each substance per 1 l of medium. The inoculation was carried out in the center of a 100 mm diameter Petri dish, in the form of mycelium excision 5 × 5 mm (*Phytophthora*), or in similar sized inoculation area created by a microbiological loop (*Pectobacterium*). During incubation, the dishes were periodically photographed to fix the area of the growing colonies of pathogens with its futher digitization using graphic analysis in the computer program Adobe Photoshop CS2. An estimation of the area (*S*) was carried out in three replications for the control (PDA) and each experimental variant with different concentrations of PPP in all types of the hydrogels. A comparative measurement of the area *S* was performed when the colonie’s sizes in PDA reached the area of the Petri dish (*S*_c_). The dimensionless *X* = (*S* − *S*_0_)/(*S*_*c*_ − *S*_0_) indicator adjusted for the inoculation area (*S*_0_) was used to calculate the median or half-maximal (50%) effective concentration (*EC*_50_) of the PPP, when *X* is equal to 0.5, as well as the effective concentration (*EC*_95_) of the complete (95%) growth inhibition, when *X* = 0.05, on the basis of the exponential model obtained in^8^:

1 $$C=A\cdot \exp (-\,kX),$$

where *C* is the concentration of the PPP, [ppm]; *A*, [ppm], *k* [dimensionless] are the empirical parameters of the model. The corresponding formulas for the calculation look like:

2 $$E{C}_{50}=A\cdot \exp (-\,0.5k);$$

3 $$E{C}_{95}=A\cdot \exp (-\,0.05k).$$

#### Water-retention, dispersity and hydraulic conductivity analysis, and technological modeling

A modified thermodynamic analysis of water-retention characteristics (WRC) and dispersity of the gel’s compositions was carried out by combination of equilibrium centrifugation^11^ with a new method of the soil water thermodesorption^12^. We used a high-speed laboratory centrifuge Hettich Universal 320 (Germany) and its Russian analogue CLN-16 with a water-retention energy range (absolute values of the soil water potential (Ψ) or the equivalent soil water pressure from 0 to 3030 J/kg (kPa) as a function of soil water content per dry weight (*W*). After the last stage of centrifugation (12000 rpm), the samples were placed for differentiated drying at temperature of 30, 40, 50, 60, 70, 80, 105 °C into a KD 200 drying oven (China) with forced air circulation and ventilation. This simple procedure estimates WRC in the range of absolute values of the thermodynamic potential of soil water from 3020 to 1000000 J/kg, as well as a specific surface area according to our methodical developments^12^. A specific surface area (*S*) of the gel compositions was evaluated in two independent ways – according to the theory of Brunauer-Emmett-Teller (*S*_*BET*_) following the transformation of WRC to desorption isotherms^12^, and directly by the slope of WRC in semi-logarithmic coordinates (*S*_*WRC*_) using the fundamental ion-electrostatic model of disjoining pressure, as proposed by^13^. This model, as a rule, adequately describes the sorption section of WRC at absolute values of the water potential of more than 1000 J/kg.

Approximation of WRC experimental data in the range of dominance of capillary forces has been performed by the van-Genuchten model^14^. The model parameters were also used to convert WRC to the structural curves pore volume distribution by the size of their radii (*V(r)*) according to the formula^15^:

4 $$V(r)=(n-1)({W}_{s}-{W}_{r}){\rho }_{b}{(1+{(\alpha P)}^{n})}^{-m-1}{(\alpha P)}^{n},$$

where *P* is an absolute value of the soil water pressure (potential); *W*_*r*_ is the residual water content corresponding to tightly bound water; *W*_*s*_ is the water content in a state of saturation of the soil (full water capacity); *α, n, m* = 1 − 1/*n* are the van-Genuchten empirical constants*;* ρ_*b*_ is the soil bulk density.

Evaluation of the values of field water capacity (*FWC*), wilting point (*WP*) and the available soil water range (*AWR* = *FWC* − *WM*) from WRC data was implemented by the Voronin^16^ method using our computer algorithm^15^. Saturated hydraulic conductivity (*K)* was determined at the laboratory permeameter in cylindrical columns 10 cm long with water supply to the surface of the sample by a Mariott device with an automatic mode of non-pressure flow.

Experimental data of water retention parameters *W*_*s*_*,W*_*r*_ *α, n* in the standard van Genuchten function as well as a saturated hydraulic conductivity *K* were used in HYDRUS-1D computer software for the technological modeling of water exchange and root consumption in a soil-gel-plant system, according to^6^. For this purpose, we used multivariate computer simulations in the HYDRUS-1D software for a potato culture with potential water consumption of 3–5 mm/day (the upper boundary condition) and with localization of the hydrogel and roots within a 20 cm soil layer in a 2 m soil profile initially saturated with water under free outflow from the lower boundary (the seepage face lower boundary condition).

#### Laboratory pathogen’s diagnosis and chemical analysis

The potential infection of tubers by pathogenic microflora was determined in the Department of Potato and Vegetable Diseases at the All-Russian Research Institute of Phytopathology (http://vniif.ru). The accepted methods was used which implies an initial 4-week incubation of pre-moistened tubers in hermetically sealed plastic bags to create optimal conditions for the development of putrefactive microflora. Species composition of the putrefied microflora pathogens were then determined. Also, for the anti-pathogenic effectiveness of the SGS in the analysis of tubers, we applied a new development in matrix PCR diagnostics of the main potato pathogens. This express analysis was performed by GenBit LLC (http://genbitgroup.com) and the All-Russian Research Institute of Phytopathology (http://vniif.ru) using the AriaDNA microarray amplifier (“Lumex” LLC, Russia) and Potato DNA Pathogens microarrays for the presence of the following bacterial and oomycetic pathogens: *Phytophthora infestans, Pectobacterium atroseptumptum, subsp. carotovorum, Dickeya dianthicola, Dickeya solani, Clavibacter michiganensis subsp. sepedonicus, Ralstonia solanacearum*. Total DNA from the potato samples (100 mg of stolon and pulp tissue from each tuber) was isolated by the method of affinity sorption using the “RIBO-sorb” isolation kit (http://InterLabService.ru). After isolation, a DNA-containing buffer solution (1.2 μl) was placed in the microarray reaction zone under a layer of mineral oil (620 μl), using a similar RNA-buffer solution without DNA as an untreated control. The filled microarrays were placed in the fuser of the AriaDNA microarray amplifier. The analysis was carried out automatically using the Ariadna software with real-time recording of the amplification results. A change in the fluorescence level corresponding to the presence of DNA of the diagnosed pathogens was displayed on the amplification graphs. The result of the analysis for the presence of the target pathogen was considered positive if the threshold cycle (*C*_*t*_) values for the corresponding pathogen do not exceed 35 units. The calculation of threshold cycles is performed by the software of the amplifier in automatic mode.

The silver content in the SGS, phytomass, soil, and drainage water solutions was evaluated by potentiometry using the Ecotest 2000 ionomer with ion-selective electrode XC-Ag-001 (Russia, http://econix.com), and by XRF-technology using the Olympus Vanta Pro analyzer (https://olympusxrf.com).

All laboratory experiments were carried out with 3–4 replicates and subsequent statistical and mathematical analysis of data using standard functions of MS Excel 2007 and the nonlinear Regression Wizard of S-Plot 2001 software.

### Field experiments

#### Potato growing experiments

The field experiments testing a new system of protection and optimization of potato rhizospheres were carried out at 3 localities in humid and arid climatic conditions, natural rainfall, and irrigation. At all sites the complete randomized block design included several different treatments and an untreated control, each in triplicate. The experimental unit with individual treatment consisted of a 2–5m-wide by 10–20m-long plot separated from adjacent plots with a 0.5–1m-wide buffer strip. The basic test site was placed in the Serebryanoborsky experimental station of the Institute of Forest Science, Russian Academy of Sciences (N55.77090274, E37.39450062) in Moscow on medium-cultivated sod-podzolic loamy sandy soils underlain by sandy alluvium. The first experiment was conducted here in the summer of 2017 under natural rainfall. The Red Scarlett potato cultivar susceptible to late blight was tested in two seed options: one was normatively free from pathogens super elite tubers, the other was highly (40–50%) infected tubers with a complex of pathogens (ordinary scab – *Streptomyces scabies*, anthracnose – *Colletotrichum coccoides*, fusariose – *Fusarium sp*., rubber rot – *Geototrichum candidum*, bacterial rot – *Pectobacterium sp*.). As controlled-release carriers, two types of SGSs (A11 and A22) were tested in randomized 50 m^2^ plots with doses of 0.5 l and 1.0 l per potato bush without mixing with the soil and with the next PPP concentrations: fungicide “Quadris” – 20, 100, 500 ppm; ionic silver – 20, 100, 500 ppm; silver nanoparticles – 20, 50, 200 ppm. The next experiment at the same locality and with the same potato variety was carried out during the growing season of 2018 in a closed polycarbonate greenhouse with automatic ventilation and 8 automated drip irrigation lines controlled by GA-319N electronic timers (China) for starting and stopping water supply and Equatel-SVK-15G flowmeters (Russia). The experiment included the following treatments: untreated control (without SGS, watering due to moisture deficit, or 100% watering time), A22 hydrogel composition with fungicide “Quadris” (50 ppm), A22 hydrogel composition with silver ions (100 ppm), and A11 hydrogel composition with silver nanoparticles (100 ppm), in doses of 0.5 and 1.0 l hydrogels per potato bush, and an SGS arrangement as a continuous layer without mixing and with uniform mixing of the hydrogel and the soil in equal volumes. The experimental plots had two drip irrigation treatments: normal (100% watering time) and economical (50% watering time relative to untreated control). A similar experiment in a closed greenhouse with manual sprinkling irrigation was carried out in 2018 farther south in semi-arid conditions at the experimental station of the Kuban State Agrarian University in Krasnodar (N45.05099734, E38.92116193). The treatment and tested compositions with PPP were the same as in the previous experiment, excluding synthetic fungicide. The Lady Claire potato variety was grown on light loamy ordinary chernozem and its compositions with SGS. The final experiment in 2018 was carried out on the Gala potato variety in arid conditions of Uzbekistan (Tashkent region, Qumaryk farm (N 41.06613390, E 69.33949355)). The experiment was conducted on typical medium loamy grey soil (serozem) under furrow irrigation. Here we tested gel compositions A11 and A22Ag, which contained 25–50 ppm silver ions per mass of 1:100 swollen SGS in two doses of 0.5 and 1.0 l for every 0.5 linear meters of the furrow, under furrow irrigation of 50 and 100% of the applicable irrigation rate.

The field work included mechanical treatment of the site (plowing, harrowing, cutting furrows), SGS embedding and planting potatoes, periodic weeding and loosening (4–8 times per season), hilling, irrigation (for irrigation treatments), pre-harvest cutting of potato tops, harvesting with a differentiated assessment of the quantity, weight, and linear dimensions (length, width) of the potato tubers. During the growing of potatoes, we carried out a morphometric manual control of the height (*H*) of the bushes and the diameter (*D*) of their projected cover. Additionally, we carried out an automated control of atmospheric and soil hydrothermal parameters (temperature, relative humidity, precipitation) and soil moisture (water-air index) in the surface (0–5 cm) layer and in the rhizosphere (10–20 cm) in accordance with previously developed methods, criteria and standards^17^. The kinetics data of potato plant growth were fitted by the Verhulst-Pearl logistic model:

5 $$H/{H}_{max}\,or\,D/{D}_{max}={\{1+a\cdot \exp (\mbox{--}bt)\}}^{\mbox{--}1},$$

where *t* is time, *H*_*max*_ and *D*_*max*_ are limit (maximum) values of the height and diameter of the potato plants during their growth period; *a, b* are the empirical constants of the model, and *b* is the so called Malthusian growth parameter. The model (5) allows us to estimate half-life characteristics (*T*_0.5_) of different treatments, according to the following simple formula:

6 $${T}_{0.5}=\,\mathrm{ln}(a)/b;$$

Hydrothermal parameters and soil moisture were estimateded in an automatic mode by programmable sensors “Hygrochron” DS1923 (https://www.maximintegrated.com/) and loggers Decagon with sensors 5TE (https://decagon.com).

#### PPP leaching experiment

The environmental risk of PPP leaching from the soil treated by plant protective acrylic SGS was assessed at the first (base) test site with the most water-permeable sandy loam soil using the most mobile SGS component in a form of silver ions. We used both the indirect method, taking into account the balance of silver in the rhizosphere, and the direct assessment of the silver ions leaching by percolated water in lysimetric soil monoliths, according to^17^. A monolith of loamy sandy soil (untreated control) or its composition with the SGS containing the same amount (100 ppm) of silver ions was placed in 50-cm PVC tube, which has a removable container-lysimeter at the bottom for the accumulation of percolated water. A PVC tube was placed in a vertical soil well with a porous casing made of foamed polyethylene. Every ten days a water sample was removed from the container and then analyzed in the laboratory for silver ion content. Using an alternative indirect method, we investigated the change in silver reserve in a 20 cm soil layer with protective SGS over a 2-year period.

## Results and Discussions

### Laboratory analysis of SGS protective antimicrobial properties

**Figure 1:**
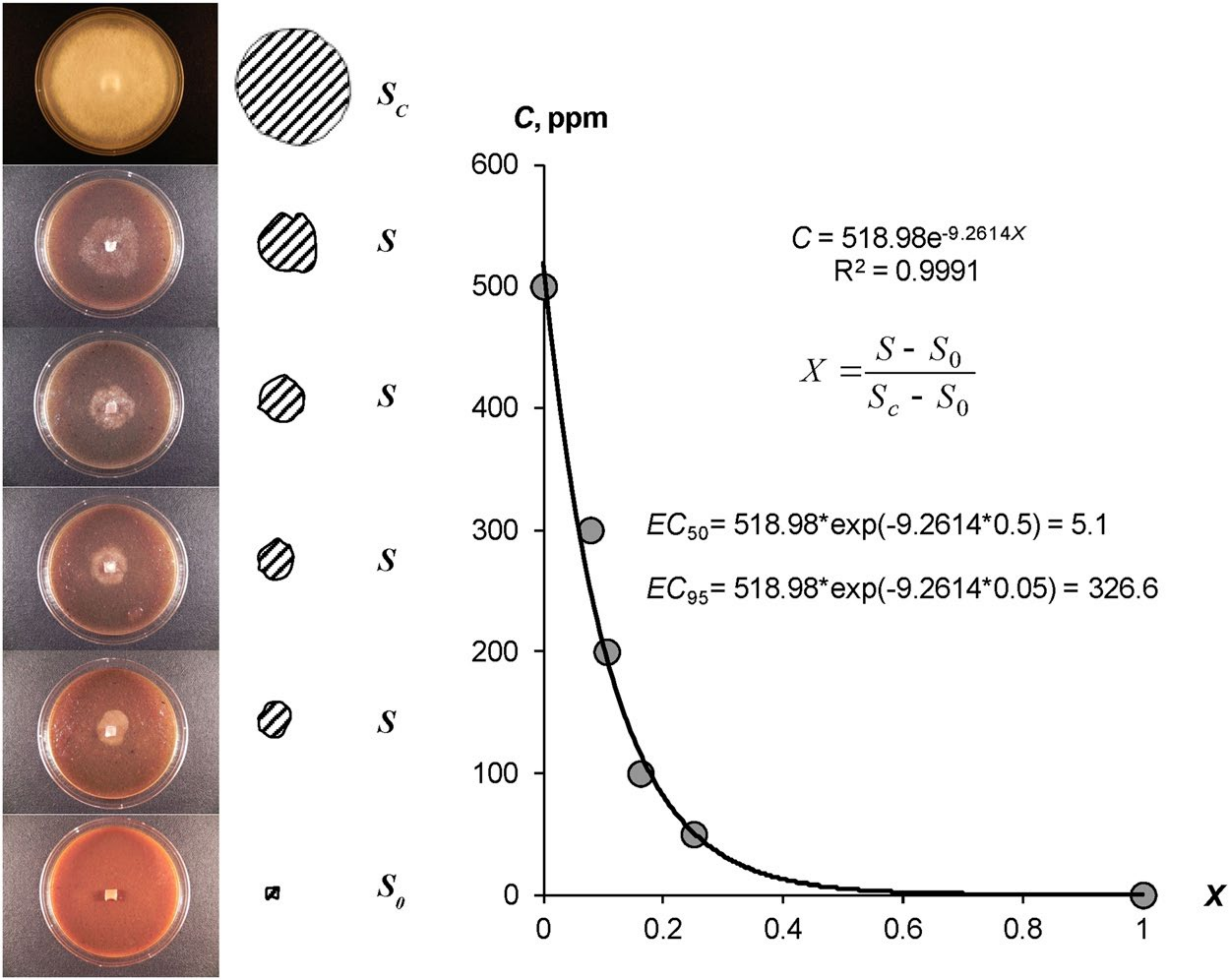
Quantitative approach to the *EC*_50_ and *EC*_95_ assesment: on the example of *Phytophthora infestans* growth in Petri dishes under the influence SGS A22 with silver nanoparticles (designations see in “Materials and methods”, formulas 1–3).

Figure 1 illustrates the visual anti-fungal effect of the composition A22 with silver nanoparticles as well as our new method for *EC*_50_, *EC*_95_ estimation using the exponential model^8^. The estimated *ЕС*_50_ and *ЕС*_95_ values for different SGS are shown in Table 1. The *EC*_50_ of the studied SGS varied within a range of 0.9 ± 0.1 to 63.0 ± 19.6 ppm. Similar results were obtained from the same laboratory microbiological method (silver ions or nanoparticles in the composition of dense, gel-like agar medium, or in the foams as saponins) in the following studies: *EC*_50_ from 30 to 50 ppm in^18^; diapason *EC*_50_– *EC*_99_ from 10 up to 111 ppm in^19^; *EC*_50_– *EC*_99_ from 50 to 100 ppm^20^; *EC*_50_ from 13 to 50 ppm^21^; *EC*_50_ from 10 to 15 ppm^22^. Gel compositions based on silver nanoparticles in the case of late blight had lower-range average values (1–17 ppm). Similar compositions with ionic silver and synthetic fungicide were characterized by higher *EC*_50_ values of 17–60 ppm relative to the late blight, i.e. lower fungicide effect. With respect to *Pectobacterium*, gel compositions based on ionic silver appeared to be generally more efficient than silver nanoparticles and synthetic fungicide. The *EC*_50_ concentrations for the hydrophilic “Aquasorb” and A11 gels varied from 1.1 ± 0.5 ppm to 5.1 ± 1.9 ppm, while the values for the compositions with nanoparticles and “Quadris” were higher, ranging from 2.4 ± 0.4 to 11.0 ± 3.5ppm. In contrast to the hydrophilic SGS, the bactericide effect of the composition with amphiphilic composition A22, silver nanoparticles and synthetic fungicide was 1.3–2 times higher than the ionic silver analogue. Therefore, the type of SGS (hydrophilic or amphiphilic) may affect the biocidal properties of SGS, which is confirmed by LSD method at *p* = 0.05 (*Phytophthora*) and *p* = 0.01 (*Pectobacterium***)** significance levels. Complete inhibition of pathogenic agent activity *EC*_95_ ranged from 187 ± 13 to 448 ± 31 ppm in the case of late blight and from 76 ± 14 to 501 ± 61 ppm for potato black leg. These values exceeded the estimations of the PPP efficiency 10–50 times or more based on the *EC*_50_ alone. The obtained *EC*_95_ values were rather higher than doses of 75–100 ppm of aquatic solution or dispersion of silver with 99% suppressive effect^9,23^. This may be due to both, slightly different mobility of PPP in aqueous solutions and gels, and the specificity of the tested microflora in aquaculture.

**Table 1:** The effective concentration for two-fold and total suppression of pathogenic microflora growth.

SGS/*patogen*/treatment	*ЕС*_50_	*ЕС*_95_
**1**. ***Phytophthora***		
**Ionic silver**		
“Aquasorb”	42. ± *4.4	448 ± 31
A11	63.0 ± 19.6	467 ± 29
A22	17.0 ± 5.4	406 ± 44
**LSD**_0.05_*	**24.0**	**70.6**
**LSD**_0.01_*	**36.4**	**106.9**
**Silver nanoparticles**		
“Aquasorb”	1.4 ± 0.2	264 ± 13
A11	0.9 ± 0.1	272 ± 80
A22	5.8 ± 2.4	241 ± 90
**LSD**_**0.05**_	**2.8**	**139.9**
**LSD**_**0.01**_	**4.2**	**211.8**
**“Quadris” sythetic fungicide**		
“Aquasorb”	14.1 ± 3.3	237 ± 18
A11	4.9 ± 3.1	222 ± 10
A22	17.3 ± 6.5	187 ± 13
**LSD**_**0.05**_	**9.1**	**28.1**
**LSD**_**0.01**_	**13.9**	**42.6**
**2**. ***Pectobacterium***		
**Ionic silver**		
“Aquasorb”	1.1 ± 0.5	63 ± 15
A11	5.1 ± 1.9	76 ± 14
A22	40.3 ± 14.0	501 ± 61
**LSD**_**0.05**_	**16.3**	**74.3**
**LSD**_**0.01**_	**24.7**	**112.6**
**Silver nanoparticles**		
“Aquasorb”	2.7 ± 0.5	341 ± 48
A11	2.4 ± 0.4	335 ± 21
A22	21.0 ± 9.3	457 ± 64
**LSD**_**0.05**_	**10.6**	**95.5**
**LSD**_**0.01**_	**16.3**	**144.7**
**“Quadris” sythetic fungicide**		
“Aquasorb”	6.0 ± 2.3	69 ± 26
A11	11.0 ± 3.5	78 ± 28
A22	28.2 ± 10.3	382 ± 59
**LSD**_**0.05**_	**12.8**	**81.2**
**LSD**_**0.01**_	**19.4**	**122.9**

*Hereinafter, ± means the boundaries of the confidence interval at *p* < 0.05 significance level, **LSD**_**0.05**_ and **LSD**_**0.01**_ are the Least Significant Differences at *p* = 0.05 and *p* = 0.01 significance levels, respectively.

The “Quadris” synthetic fungicide has a half-life of 3 ≤ *T*_0.5_ ≤ 39 days (http://wineryclub.info/handbook/14-kvadris.html). This obviously guarantees its total biodegradation in our gel compositions with a half life of at least 3–5 years, according to^4^. In contrast to these biodegradable PPP, the silver-gel compositions require an assessment of maximum allowable concentrations. For aquatic plants and aquaculture, a two-fold decrease in growth occurs at 10–20 ppm (up to 150 ppm for green algae) of silver ions or nanoparticles^24,25^. In the soil, for which protective SGS are designed, the value *EC*_50_ for plants varies in the range of 50–1000 mg/kg of the solid phase^26^. In terms of the soil liquid phase, it gives the range from 250 to 5000 ppm at 20% water content. Shclich *et al*.^27^ reported a slightly smaller *EC*_50_ for ionic and colloidal silver ranging from 200 to 400 ppm for soil earthworms after experiments with silver doses of 15 to 1000 mg/kg or from 75 to 5000 ppm at 20% soil moisture, respectively. Taking into account the obtained results, we have chosen to test a PPP range of 20 to 500 ppm in the field.

### Thermodynamic laboratory analysis of WRC, dispersity, and saturated hydraulic conductivity of SGS

**Figure 2:**
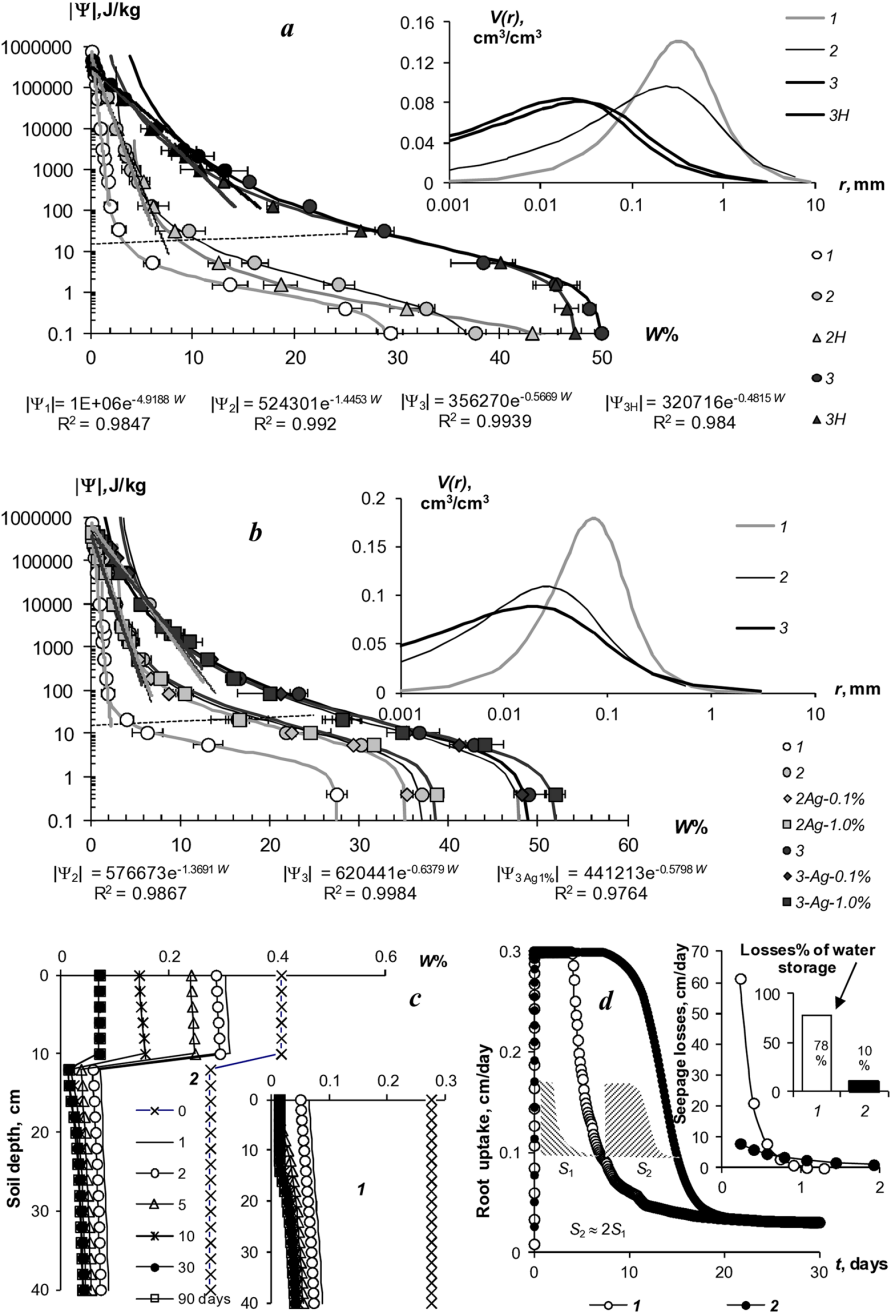
WRC of synthetic gel structures and technological modeling of their application in the soil: (**a**,**b**) WRC (main figure), pore distribution (inset); *1* – control, *2* – 0.1%, *3* – 0.3%SGS, *H* – humates; (**c**) water regime modeling, (**d**) root water uptake and down flow losses modeling: *1* – sandy soil, *2* – 0.2%SGS.

The water-retention characteristics of several SGS and the structural curves of pore size distribution calculated by WRC are shown in Fig. 2 for A11, A11H (*a*), and A22 (*b*). The solid lines in the main figures of the WRC represent the fundamental ion-electrostatic model of disjoining pressure^13^ and the standard van-Genuchten model^14^; the dotted line shows the secant for determining the field water capacity by the Voronin^16^ method. All tested compositions were characterized by a stable increase in water-retention proportional to a dose of the hydrogels over a wide 0–1000 kJ/kg range of the absolute soil water potential values. The WRC of the tested SGS have been regularly shifted right with respect to the control position (mineral substrates), which indicates an increase in energy of water-retention and moisture capacity of the samples. Alternitavely, pore size spectra had a tendency of moving left towards smaller sizes. Such tendency, we believe, reflects an effect of aggregation of the mineral mass by SGS. Table 2 contains traditional agrophysical parameters of field water capacity, the available soil water range and the indicators of a specific surface area estimated by the BET method and by the slope of WRC. Field water capacity at hydrogel doses of 0.2–0.3% from the soil dry mass constitutes a 15–25% compared to a 3.3–4.6% in sandy substrates, which means that water retention in such doses increases 5–8 times corresponding to translation of the original sandy substrates in the loamy soil by this indicator. Along with that, the increase in the wilting point rarely exceeded 2–5 times. As a result, the *AWR* was expanding 4–8 (up 10–13) times as compared to the original mineral substrates (Table 2). A specific surface area of the studied SGS increases 3–3.8 times at hydrogels concentrations of 0.1% and up to 6.0–10.2 times at higher doses of 0.2–0.3%, reaching the values of 40–60 m^2^/g (up to 75 m^2^/g), typical for light and medium loams. From a methodological point of view it’s important to note that the specific surface area estimated by the standard BET method actually coincided with one estimated by the slope of WRC proposed in^13^ on the basis of the fundamental ion-electrostatic model of disjoining pressure. The usage of amphiphilic fillers (humates, peat) in the production of the Ural Chemical Factory did not significantly change water retention properties of the SGS compared to hydrophilic formulations of A11 and “Aquasorb”. A similar result concerning insignificant (at *p* = 0.01 significance level according LSD_0.01_ criterion) changes in water retention is obtained by comparing the amphiphilic options supplemented or not with electrolyte’s admixtures (A11HMZ vs A 11H). Inclusion of ionic groups of electrolytes in the acrylates and humates form in the structure of a polymer matrix allows us to solve the problem of reducing the hydrogel’s swelling as affected by the osmotic stress^28^. Moreover, embedded cations can react in ion exchange and serve as a source of elements of plant’s mineral nutrition. As the experiment with the samples of A22 Ag amphiphilic gel at a dose of 0.1–1% silver shows, the compositions based on it did not significantly worsen water retention, dispersion and structural properties, which remained at the level of “Aquasorb” European brend in the same SGS concentrations of 0.1–0.3% (Fig. 2a,b; Table 2). All obtained data of the WRC have been fitted adequately (determination coefficient R^2^ = 0.98–0.999, relative standard approximation error s = 2–7%) by the standard van Genuchten model^14^ in the capillary range (|ψ| = 0–1000 J/kg) and by the fundamental ion-electrostatic model^13^ in the range of surface absorption mechanisms (|ψ| > 1000 J/kg), as the Fig. 2a,b shows. The parameters of the van Genuchten model along with the values of saturated hydraulic conductivity (*K*) are contained in Table 2. The use of the SGS greatly (up to 10–80 times) reduces water permeability of sandy soil substrates that, along with an increase in water retention, should minimize unproductive water losses from the rhizosphere. The next section confirms this assumption using a computer simulation of water exchange in a soil-gel-plant system.

**Table 2:** Estimated parameters of water retention, conductivity and dispersity in mineral-gel compositions.

SGS doses %	FWC%	WP %	AWR%	Ws m^3^/m^3^	Wr m^3^/m^3^	*α* kPa^−1^	n	K cm/day	*S*_*WRC*_ m^2^/g	*S*_*BET*_ m^2^/g
**Monomineral quartz sand (control-1):**										
0%	4.6	1.3	3.3	0.27	0.01	0.31	2.26	520	7.4	8.0
**“Aquasorb” SGS**										
0.1%	19.2	4.2	15.0	0.39	0.03	0.146	1.61	29	27.4	31.0
0.2%	26.0	8.3	17.6	0.49	0.04	0.209	1.40	17	47.8	51.1
0.3%	28.5	9.8	18.7	0.49	0.01	0.185	1.30	9	64.0	63.6
**LSD**_**0.05**_	**2.6**	**0.8**	**1.8**	—	—	—	—	**31.3**	**5.1**	**5.3**
**LSD**_**0.01**_	**3.9**	**1.2**	**2.7**	—	—	—	—	**47.4**	**7.7**	**8.0**
**A22 SGS**										
0.1% РМ	18.6	4.0	14.5	0.37	0.01	0.183	1.50	37	26.5	26.9
0.1% + Ag 0.1%	17.0	4.1	12.9	0.35	0.03	0.169	1.61	—	28.2	30.0
0.1% + Ag 1%	19.5	4.1	15.3	0.39	0.01	0.199	1.45	29	26.0	23.3
0.2%	23.7	5.8	17.9	0.41	0.02	0.13	1.44	19	36.1	35.0
0.2% + Ag 0.1%	22.7	6.6	16.1	0.40	0.02	0.188	1.36	—	42.5	45.1
0.2% + Ag 1%	21.6	6.9	14.6	0.39	0.06	0.118	1.63	17	44.4	42.3
0.3%	28.6	9.5	19.1	0.49	0	0.203	1.29	6	56.8	55.0
0.3% + Ag 0.1%	27.4	9.9	17.5	0.48	0.03	0.232	1.32	—	53.5	55.2
0.3% + Ag 1%	29.0	9.6	19.4	0.52	0.03	0.213	1.34	10	62.5	61.9
**LSD**_**0.05**_	**2.7**	**0.8**	**1.9**	—	—	—	—	**23.7**	**5.0**	**5.0**
**LSD**_**0.01**_	**4.1**	**1.2**	**2.9**	—	—	—	—	**35.9**	**7.6**	**7.5**
**Monomineral quartz sand (control-2):**										
0%	3.3	1.7	1.6	0.29	0.02	1.655	1.87	460	7.1	8.0
**A11 SGS:**										
0.1%	10.2	3.6	6.6	0.38	0.02	1.913	1.42	31	22.6	24.5
0.2%	20.7	9.6	11.1	0.53	0	6.459	1.19	15	62.7	62.9
0.3%	28.4	11.4	17.0	0.50	0.02	0.323	1.26	9	75.2	73.3
**LSD**_**0.05**_	**2.2**	**0.9**	**1.3**	—	—	—	—	**27.7**	**6.0**	**6.0**
**LSD**_**0.01**_	**3.3**	**1.4**	**1.9**	—	—	—	—	**41.9**	**9.2**	**9.1**
**A11H SGS:**										
0.1%	8.6	4.5	4.1	0.50	0.04	7.257	1.47	26	23.8	23.7
0.2%	16.7	7.2	9.5	0.49	0	9.817	1.20	17	45.7	47.0
0.3%	28.1	9.7	18.3	0.48	0	0.230	1.27	8	75.2	75.0
**LSD**_**0.05**_	**2.0**	**0.8**	**1.3**	—	—	—	—	**27.7**	**5.5**	**5.5**
**LSD**_**0.01**_	**3.1**	**1.2**	**1.9**	—	—	—	—	**41.9**	**8.3**	**8.4**
**A11HMZ SGS:**										
0.1%	9.4	4.6	4.8	0.57	0.04	7.337	1.46	31	25.6	28.2
0.2%	15.2	6.2	9.0	0.53	0.02	7.389	1.27	16	39.1	37.5
0.3%	29.7	9.5	20.2	0.53	0.02	0.231	1.29	10	56.6	59.6
**LSD**_**0.05**_	**2.1**	**0.7**	**1.4**	—	—	—	—	**27.7**	**4.4**	**4.6**
**LSD**_**0.01**_	**3.2**	**1.1**	**2.1**	—	—	—	—	**41.9**	**6.7**	**6.9**

Note: ***FWC*** is field water capacity, ***WP*** is wilting point, ***AWR*** is available soil water range; ***Ws***, ***Wr α***, ***n*** are the van-Genuchten model parameters (see formula 4); ***S***_***WRC***_, ***S***_***BET***_ are specific surface area obtained by WRC and BET methods, respectively.

### HYDRUS-1D computer modeling of water exchange and root consumption in a soil-gel-plants system

The simulation results (Fig. 2c,d) revealed a regular increase in water supply of the rhizosphere and prolongation of active root water uptake in proportion to SGS doses. The minimum doses (0.1–0.2%) of SGS in a 10 cm layer is quite enough for a 1.5–2-fold increase in available water storage within the rhizosphere relative to the initial sandy substrate and prolongation of active (3–5 mm/day) potato root consumption from 5 to 10 days. This effect occurs due to the increased water retention of the gel compositions, as well as a strong decrease (up to 10 times) in unproductive seepage losses of water from the rhizosphere through the gravitational outflow (Fig. 2d inset). A layered method for the SGS placing was more effective in reducing capillary-gravitational outflow of water from the rhizosphere compared to a uniform gel’s distribution. The total root consumption during the growing season (the area under the curves in main Fig. 2d) divided by the transpiration coefficient gave an estimate of the potential productivity of the potato when using SGS. For the tested Red Scarlett, Lady Claire and Gala potato varieties, these values were approximately 30, 40 and 60 t/hct.

Summarizing the preliminary laboratory study and the results of technological modeling, we selected for field experiments working doses of hydrogels in the range of 0.1–0.2% with PPP content from 20 to 200 (up to 500) ppm both in uniform SGS layout and in mixing with the soil. Presumably, they were supposed to ensure the planned potential productivity of the test potato varieties (30–60 t/hct) with 1.3–2 times water saving and effective crop protection from the main potato pathogens, including late blight. Subsequent field trials fully confirmed the possibility of obtaining such results in practice.

### Potato growing field experiments with SGS under atmospheric precipitation (2017)

**Figure 3:**
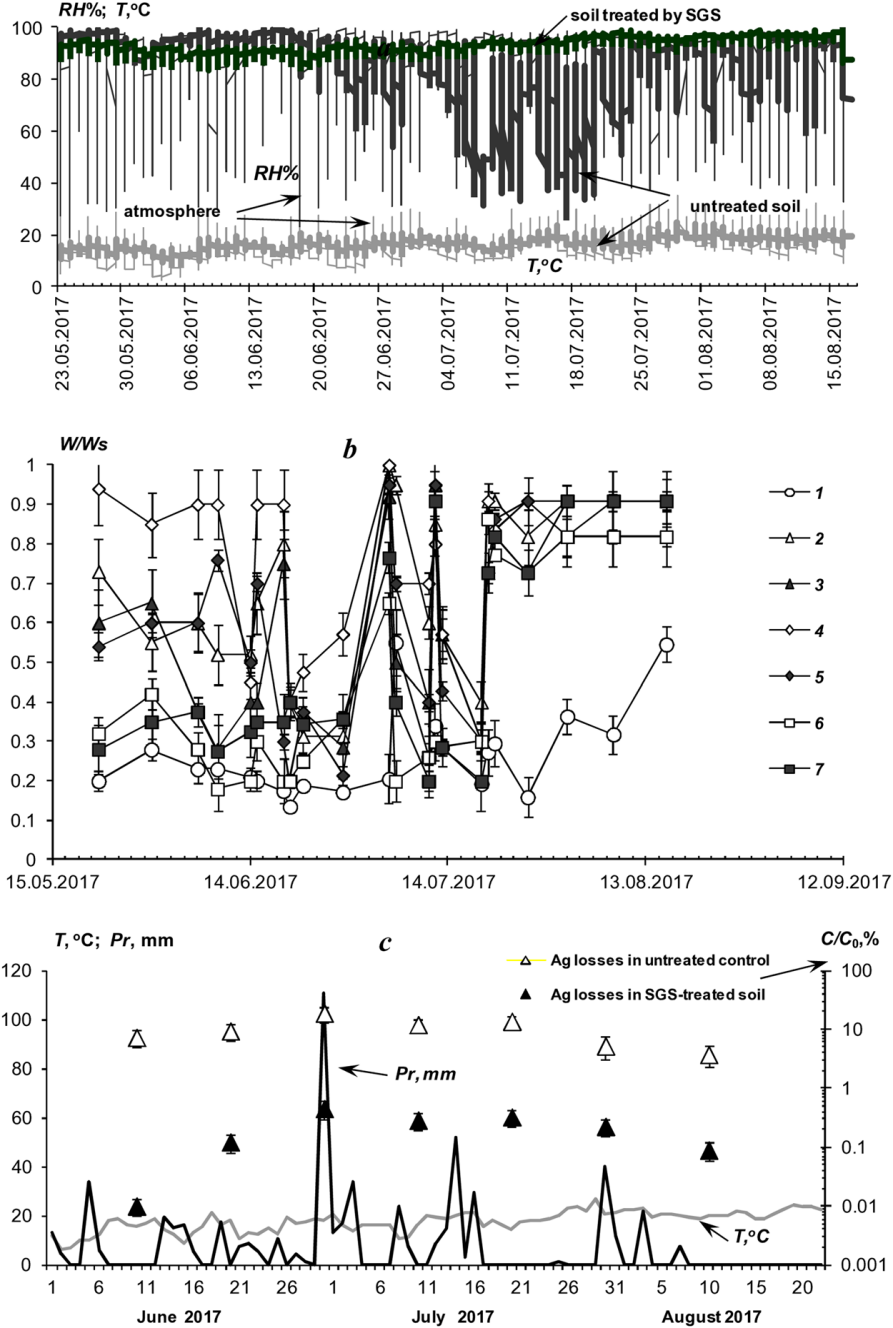
Monitoring of meteorological conditions and soil regimes in the 2017 experiment; (**a**) temperature (*T*) and relative humidity (*RH*%); (**b**) soil *W/Ws* index (*W* is soil water content per weight, *Ws* is water content at soil saturation): *1* – untreated control, *2* – CGS A11+Ag ionic, *3* – PGB A22+Ag ionic, *4* – CGS A11+Ag nanoparticles, *5* – CGS A22+Ag nanoparticles, *6* – CGS A11+“Quadris”, *7* – CGS A22+“Quadris”; (**c**) daily temperature, precipitation (*Pr*) and lysimetric removal of silver ions from the soil (*C/C*_0_): *С*_0_ is initial silver content in a 30 cm soil layer [g/m^2^], *С* is leached silver per decade [g/ m^2^].

**Figure 4:**
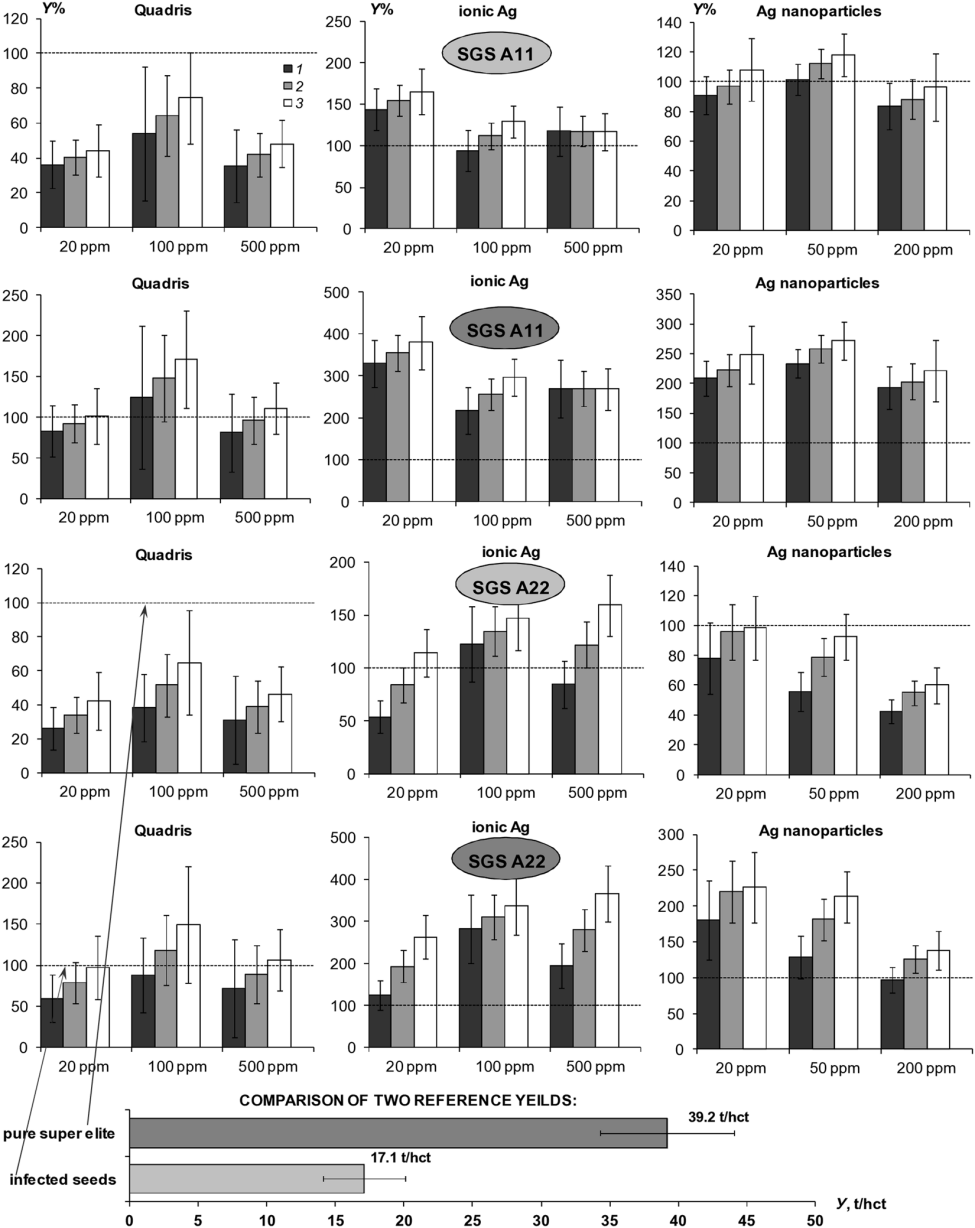
Comparison of the results of potato yields under the influence of SGS (2017 experiment); SGS dozes per potato bush: *1* – 1.0 l, *3* – 0.5 l, *3* – the average yeld between *1* and 2.

The first field experiment was held in the European part of the Russian Federation in 2017 under difficult weather conditions with excessive precipitation and widespread development of late blight. An example of the results of monitoring hydrothermal indicators: temperature (*T*), relative humidity (*RH*%), as well as the index of the water-air regime of the soil (*W/Ws*, where *W* is the soil water content; *Ws* -water content in saturated state) is shown in Fig. 3. A ratio W/Ws close to 0 or 1 means the lack of water or air in the plant rhizosphere, respectively^17^. During the growing season, temperatures ranged from 2.6 to 35.1 °C with an average of *T* = 17.0 ± 6.0 °С, for the entire period, and relative humidity from 19.4 to 100% with an average value of *RH*% = 78.8 ± 20.5%. This indicates a high probability of developing a moisture-loving pathogenic microflora, including late blight. In the potato rhizosphere treated by the SGS, the *RH%* ranges from 85 to 100%, and the *W/Ws* ratio from 0.2 to 1 with the dominance of optimal values of *W/Ws* = 0.6–0.9. In the untreated control and in the upper soil layer over the hydrogel compositions, these indicators were significantly lower: *RH*% = 31–100% and *W/Ws* = 0.1–0.6. The common trend of a 1.5-3-fold excess of the *W/Ws* index for the layer with hydrogel compositions compared to the untreated control indicated a similar increase in the amount of available water and, accordingly, potential potato productivity. In general, despite unforeseen extreme weather conditions and an increased infectious background, we managed to achieve a 1.3-2-fold increase in potato yield with 100% protection of tubers from a massive outbreak of late blight (Fig. 4). In the plots with the initially infected (40–50%) seeds, the excess of the crop was 100–200% relative to the untreated control (a reference yield of 17.1 ± 8.6 t/hct). The SGS teeated plots with initially healthy uninfected seeds had a 20–60% increase a reference yield of 39.2 ± 4.9 t/hct in the untreated control. An assessment of the potential pathogenic affection of potato tubers after their incubation in plastic bags gave no more than 4–5% damage by epiphytic microflora (ordinary scab) for the SGS protective treatment, whereas on the untreated control, pathogenic damage reached 80–100% with deep infection penetrating into tubers due to development of fungal and bacterial rots (*Phytophthora infestans*, *Colletotrichum coccoides*, *Fusarium sp*., *Streptomyces scabies, Geototrichum candidum*, *Pectobacterium sp*.). Alternative PCR diagnostics of freshly harvested tubers revealed the presence of pathogens: *Phytophthora infestans, Clavibacter michiganensis subsp. sepedonicus, Pectobacterium atrosepticum and Pectobacterium carotovorum subsp. carotorum* in the untreated control, however any target potato pathogens were not detected on the tubers from the SGS treated plots. A visual assessment of the SGS protective treatments did not exceed 1–2% of actual pathogenic damage of potato tubers of a new yield, while the overhead potato phytomass was strongly affected by late blight: 100% in the untreated control plots, 80–100% in the case of compositions with silver nanoparticles, 30–50% for the ionic silver and 10–30% for the “Quadris” fungicide compositions, which, in contrast to silver nanoparticles, demonstrated a translaminar effect able to protect the aerial parts of plants.

The morphometric measurements revealed a statistically significant increase (up to 1.5–2 times) of root length under the influence of the SGS, with the exception of the “Quadris” fungicide compositions, a close correlation between the height and the projected diameter of the potato bushes (*D* = 0.913 *H*, R^2^ = 0.83, for 943 plant’s samples) and the full correspondence of the potato growth to the Verhulst-Pearl logistic model (R^2^ = 0.98–0.99) with the Malthusian parameters *b* = 0.11–0.17 day^−1^, half-life *T*_0.5_ = 30–39 days, *H*_*max*_ = 22–53 cm. The amphiphilic compositions A22 were found to be most effective in conditions of an abnormally wet year at medium silver-based PPP concentrations (100 ppm) and low SGS application doses of 0.5 l/bush. High (400–500 ppm) doses of silver in the protective SGS, and in the case of the “Quadris” fungicide, the relatively low concentrations (20–100 ppm) can inhibit the growth of the plants themselves and the yield of potato tubers. However, a clear advantage of the “Quadris” fungicide composition is a translaminar effect because of which only this treatment has been resisted complete destruction of the overhead potato phytomass by late blight in the second half of the summer. Ionic silver also had a translaminar effect, which was first proved by a quantitative pointwise (local) analysis of the silver content in tops, tubers, and green berries of potato using non-destructive XRF-technology. This effect delayed the development of late blight of vegetative organs in treatments with ionic silver for a week, compared with the untreated control and the SGS containing silver nanoparticles, apparently not capable of active root uptake and transport within the plant. Scanning layer-by-layer XRF analysis (penetration depth not more than 1 mm) showed silver accumulation only on the surface of the tuber peel (up to 30 ppm), in the absence of more deep penetration, whereas in the potato berries it was distributed almost uniformly. The presence of a protective coating of silver on the surface of the tubers, apparently, should be a significant factor that preserves the tubers from rotting during their storage^24^.

### Potato growing field experiments with SGS under irrigation (2018)

**Figure 5:**
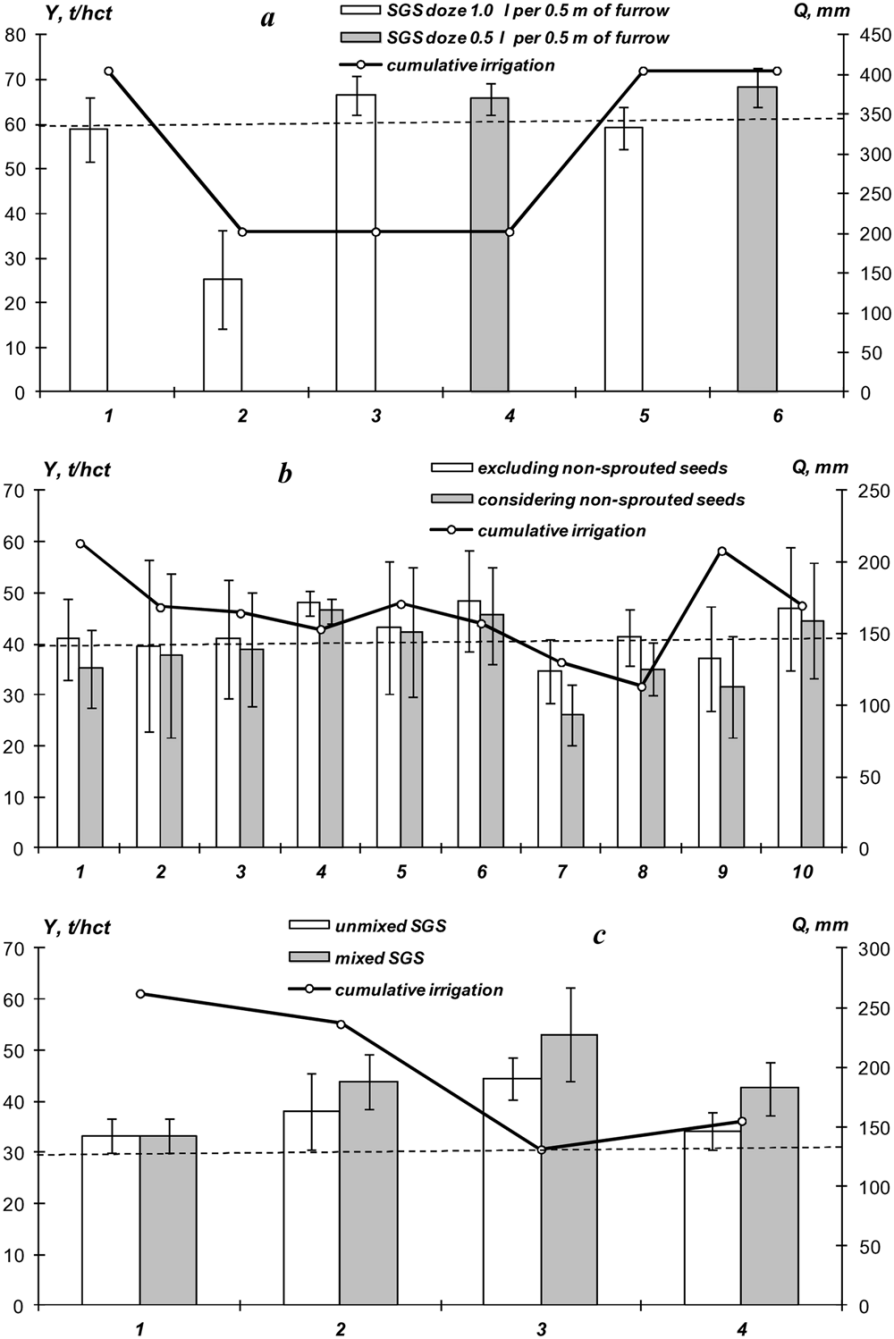
Comparison of the results of potato yields under the influence of SGS and irrigation (2018 experiments); (**a**) furrow irrigation in Uzbekistan: *1, 2* – untreater control 100 and 50% irridation; *3, 4* – A22Ag SGS 50% irrigation; *5* – A11 SGS 100% irrigation, *6* – A22Ag SGS 100% irrigation; (**b**) sprinkler irrigation in Krasnodar: *1* – untreater control, *2-6* – A22 SGS 1.0 and 0.5 l per bush without and with mixing, *7-*1*0* – same for A11 SGS; (**c**) drip irrigation in Moscow: *1* – untreater control, *2* – A22 SGS+“Quadris” 50 ppm, *3* – A22 SGS+Ag ionic 100 ppm, *4* – A22 SGS+Ag nanoparticles 100 ppm.

The field experiments that followed in 2018 confirmed the effectiveness of water-accumulating and anti-pathogenic SGS for irrigated potato farming in open and closed (in greenhouse) soil conditions. The main experiment in Uzbekistan, conducted under 100% furrow irrigation, showed that the average harvest at the untreated control of 58.8 ± 7.3 t/hct was close to the potential of the Gala potato variety (60 t/hct). However, a two-fold decrease in irrigation was accompanied by a sharp decline in potato yields of the untreated control. The SGS application significantly increased the yield by 6.9–9.5 t/hct (Fig. 5a), as well as the height of potato bushes, size, weight, and in some cases – the density of tubers, what may be associated with a starch accumulation. The growth of the Gala potato variety also corresponded well to the logistic model (5) with the parameters: *b* = 0.21–0.35 day^–1^, *T*_0.5_ = 14–21 days, *H*_*max*_ = 43–47 cm, *D*_*max*_ = 31–38 cm. Large doses of hydrogels (1.0 l), as in the previous experiment, slowed the germination of tubers and the rate of further growth of potatoes with a difference in *T*_0.5_ of 3–6 days. The SGS options under 100% and 50% furrow irrigation treatments did not show a statistically significant difference from each other in yield (Fig. 5a). That is, an additional 50% saving of the irrigation water does not lead to a decrease in potato productivity. Matrix PCR diagnostics did not reveal the presence of any potato pathogens in tubers protected by the SGS, while the following bacteria were detected in untreated control: *Pectobacterium atrosepticum) and Pectobacterium carotovorum subsp*. A comparative analysis of atmospheric monitoring data (more than 1000 measurements with an interval of 2 hours) showed extreme changes in temperature from 3.6 to 55 °C and relative humidity from 12 to 98.5%, during the whole experiment. In the uppermost 5 cm of the soil layer, temperature fluctuations during the season amounted to an amplitude of 13.1–40.6 °C, and *RH*% values of 58–100%. The average seasonal values of temperature and relative humidity in the atmosphere were equal to 23.2 ± 9.4 °C; 42.6 ± 20.4%, and in the rhizosphere *T* = 24.1 ± 5.5 °С; *RH%* = 75.1 ± 32.4%, indicating more favorable conditions for potato growth than previous trials in a humid climate. The index of air-water regime on the untreated control often diagnosed the state of overwetting, while when using the SGS mixed with the soil, the value of W/Ws was more often within the optimum range, not exceeding 0.9 (lack of air) and not falling below 0.3 (lack of moisture). When the SGS was uniformly spread at a dose of 1.0 l per bush and under 100% irrigation treatment, overwetting of the root zone often occured. However, the tubers did not rot due to the strong potential evaporation and the high transpiration activity of plants.

Additional experiments carried out in closed ground with sprinkling and drip irrigation in conditions of the Krasnodar region and the Moscow region also allowed us to achieve the planned yield with 30–50% savings in irrigation water (Fig. 5b). The experiments were carried out under similar temperature conditions (ranges of temperature variation from 4.1 to 43.6 °C with an average value of 21.1 ± 8.8 °C in Moscow and from 13.0 to 41.0 °C with an average value of 23.4 ± 5.5 °C in Krasnodar), however in completely different conditions in regards to air humidity, which sets the transpiration of water. In the Moscow greenhouse with automatic ventilation, relative humidity varied from 20 to 100% with an average value of 80.4 ± 23.7%, and in the Krasnodar glasshouse, a closed type, it was always near the saturation state (variation 84.7–100% with an average value of 93.4 ± 3.7%). As a result, in the Moscow experiment with drip irrigation in a ventilated greenhouse, the germination of tubers and further growth occurred without any delay. The growth rate of the vegetative mass according to the Verhulst-Pearl model (5) was characterized by typical values of *T*_0.5_ = 26–32 days, slightly higher than in the Uzbek experiment, apparently as a result of shorter daylight hours. Other growth parameters (*b* = 0.15–0.22 day^−1^, *H*_*max*_ = 38–66 cm, *D*_*max*_ = 34–58 cm) characterized a more intensive growth of the tested Red Scarlett potato variety in the greenhouse compared to last year’s cultivation of the same variety in open ground and atmospheric precipitation.

In the Krasnodar experiment, the growth of vegetative mass was accompanied by a significant delay (*T*_0.5_ = 38–60 days), despite the resultant plant size (*H*_*max*_ = 37–65 cm, *D*_*max*_ = 31–52 cm). We interpret this to be a result of a decrease in transpiration activity and the associated photosynthetic rate due to constantly high air humidity of the unventilated glasshouse. This situation is confirmed by direct monitoring of the index of water-air regime of the soil, that for the rhizosphere layer in the untreated control, and in the case of hydrogel compositions with uniform application without mixing, almost constantly exceeded the value of 0.9, indicating overwetting. Only SGS treatment using the amphiphilic composition A22 at a dose of 0.5 l per potato bush with obligatory mixing with the soil was characterized by the best values of the *W/Ws* index, which are, as a rule, in the optimum range for the loamy chernozem: 0.3 < *W/Ws* < 0.9. Most likely, this was due to the loosening and structuring effect on the soil by the new composition with amphiphilic filler. Overwetting of chernozem in some cases led to rotting and loss of tuber germination (untreated control, the SGS A11 treatment with uniform application) and to the development of pathogens (*Phytophthora infestans, Pectobacterium atrosepticum, Clavibacter michiganensis subsp. sepedonicus*), that affected 2–5% of potato tubers, according to matrix PCR diagnostics. In the amphiphilic A22 SGS treatment, regardless of the dose (0.5, or 1.0 liter per bush), the method of application (uniform or with mixing) and the rate of irrigation (50 or 100%), as well as in the hydrophilic A11 SGS treatment when applied with mixing, the fresh yield did not contain any pathogenic organisms, despite the presence of pathogen DNA (*Clavibacter michiganensis subsp. sepedonicus, Pectobacterium atrosepticum*) in the tubers of the Lady Claire potato variety as an original planting material. The average yield on the untreated control (41.0 ± 8.0 t/hc) reached the potential of the Lady Claire potato variety (40 t/ha). However, it experienced strong fluctuations, and taking into account the fraction of the area on which the plants did not sprout, it was significantly lower: (35.3 ± 7.6 t/hc). Overwetting of chernozem was most negatively affected in plots with the hydrophilic composition A11, where in a number of cases the actual yield fell to a level of 26–31 t/ha. The A22 SGS treatment with amphiphilic filler was better, and here the average yield was stably higher than on the untreated control, reaching 46–48 t/ha in the options of mixing the gel with the soil (Fig. 5b).

In the Moscow experiment with the cultivation of the Red Scarlett potato variety under automated drip irrigation and ventilation of the greenhouse we obtained more effective results of crop production increase (Fig. 5c). The SGS appears to have the greatest effect in coarse-textured soils, where the initial water retention and nutrient absorption capacity are much lower than in loamy chernozems and serozems. The yield on untreated control plots (33.3 ± 1.6 t/hct) was significantly higher than the predicted level of 30 t/ha for the Red Scarlett potato variety and the options with hydrogels consistently exceeded the untreated control, reaching a 30–60% yield increase. The best results (the potato crop from 38.0 ± 7.5 to 53.1 ± 9.1 t/hct) were again demonstrated by the SGS based on the new A22 gel with an amphiphilic filler, “Quadris” synthetic fungicide (50 ppm) or ionic silver (100 ppm) as a PPP. Mixing the gel structures with the soil increased an average yield by 5–8 t/hct, but this increase was often not statistically significant, considering the large variation in yield on these plots (confidence intervals of 5–9 t/ hct at *p* = 0.01 significance level). Owing to a fully automated drip irrigation system with the ability to adjust through timers the amount of water supplied, it was possible to achieve water savings for all plots with the SGS treatments (Fig. 5c). In the “economical irrigation” options, this decrease reached 1.7–2-fold differences compared to the untreated control, that is, saving up to 50% of irrigation water not only without any damage to productivity, but also with a significant increase. This result is explained by high (50% or more) unproductive losses of irrigation water through intensive infiltration and surface evaporation in coarse-textured soils^6^. These losses are completely blocked by SGS in the rhizosphere during local drip irrigation of each potato bush. Comparative monitoring of the air-water regime index showed that it was mainly within the optimal range for sandy soils (0.15 < *W/Ws* < 0.9), and only the uniform (without mixing with soil) SGS application occasionally exceeded this range. Matrix PCR diagnostics did not reveal any pathogenic organisms either in the untreated control, or in plots with the SGS treatment, which can also be explained by good quality of seed material (harvest 2017 from experimental plots with our protective rhizospheric compositions).

### PPP leaching field experiment

A field study of the acrylamide leaching from the soil, as a possible product of biodegradation of acrylic hydrogels, clearly shows that this factor can be neglected due to the insignificance of such losses^29^. A direct lysimetric analysis of the leaching of silver ions carried out in the extremely wet year of 2017 did not reveal any significant losses from the SGS. The total seasonal losses with the infiltration runoff from the soil monolith treated by the SGS amounted to less than 1–2% of ionic silver contained in the composition (Fig. 3c). As opposed to the leaching of the same doses of silver in the untreated control without SGS was quite significant (up to 70% of the initial amount introduced into the rhizosphere in the form of dry silver nitrate). However, an alternative method for estimating the removal of silver from the rhizosphere, using an analysis of the dynamics of Ag reserves in a 30 cm soil layer over a three-year period (2017–2019), showed the possibility of more intense losses compared with the data of the lysimetric analysis. In the plots treated with SGS, the total losses were 5–8% of the initial amount of silver in the rhizosphere. It can be explained by the duration of the leaching process with nearly 5-fold excess of a direct lysimetric experiment (three months). An additional explanation includes the absorption of silver by vegetation, which is confirmed by non-destructive XRF-diagnostics of tubers, green berries and vegetative potato phytomass. One more hypothesis is the effect of preferential fluxes that are absent in small lysimeters, but quite probably can appear in large plots with sandy soils^30^. All these hypotheses should be studied in the future, however, it is now clear that the losses of PPP from the SGS is significantly (at least 30 times) less than from untreated soil, and they cannot cause any great damage to the environment.

## Conclusions

A new type of acrylic hydrogels filled by amphiphilic agents (humates, dispersed peat), silver ions, nanoparticles, and organic fungicide is an effective approach to optimizing the edaphic properties and anti-pathogenic protection of the rhizosphere. The use of synthetic gel structures in sustainable potato farming for the first time made it possible to reliably protect the soil and tubers from the most common of potato pathogens, including *Phytophthora infestans*, to obtain significant (up to 6–15 t/ha) increases in yields, and in the case of irrigation, to achieve 1.3–2-fold water savings. Synthetic gel structures reliably fix plant protection products in the soil, protecting them from leaching, which allows us to recommend these effective carriers for modern agrochemical delivery systems.

## Data Availability

The datasets analysed during the current study are available from the corresponding author on reasonable request.
